# The Comparison of The Effects of Silybin and Silybin-Phosphatidylcholine on Viability and *ESR* Expression in Human Breast Cancer T47D Cell Line

**Published:** 2014-10-04

**Authors:** Narges Mahmoodi, Nasrin Motamed, Seyed Hassan Paylakhi

**Affiliations:** 1 Department of Cell and Molecular Biology, Kish International Campus, University of Tehran, Kish, Iran; 2 School of Biology, University College of Science, University of Tehran, Tehran, Iran; 3 School of Biology, Damghan University, Damghan, Iran

**Keywords:** Silybin, Silybin-Phosphatidylcholine, Breast Cancer, *ESR1*

## Abstract

**Objective:**

Silybin is a polyphenol with anti-oxidant and anti-cancer properties. The poor bioavailability of some polyphenols can be improved by binding to phosphatidylcholine. In recent years, studies have been conducted to evaluate the anti-cancer effect of silybin. We studied the effect of silybin and silybin-phosphatidylcholine on *ESR1* and *ESR2* gene expression and viability in the T47D breast cancer cell line.

**Materials and Methods:**

In this experimental study, a 3-(4,5-Dimethylthiazol-2-Yl)-2,5-Diphenyltetrazolium Bromide test (MTT test) was used to determine doses for cell treatment, and the gene expression was analyzed by real-time reverse transcriptase-polymerase chain reaction (real-time RT- PCR).

**Results:**

Significant dose- and time-dependent cell growth inhibitory effects of silybin and silybin-phosphatidylcholine along with *ESR1* down-regulation were observed in T47D cells. In contrast to *ESR1*, the T47D cell line showed negligible *ESR2* expression.

**Conclusion:**

This study suggests that silybin and silybin-phosphatidylcholine down-regulate *ESR1* in ER^+^breast cancers. Results also show that in the T47D cell line, silybindown-regulation of *ESR1* compared with silybin.

## Introduction

The estrogen receptor α (ERα) is frequently observed to be overexpressed in breast cancer (1), and has many functions, including tumor growth enhancement, and is also a prognostic and predictive factor (2). Estrogen receptor exists in two forms, ERα and ERβ, which have distinct tissue expression patterns. ERα and ERβ are encoded by *ESR1* and *ESR2* respectively, which are found at different chromosomes (6q25.1 and 14q22-25 respectively) (3). Stimulation of transcription by ERα occurs via a number of distinct molecular events in the nucleus. ERα homo- or heterodimerizes with other nuclear receptors such as estrogen receptor β (ERβ) or androgen receptor (AR) and binds, via the DNA-binding domain (DBD), to estrogen response elements (EREs) located on the promoters of estrogenresponsive genes (4).

Silybin (silibinin), the major component of milk thistle (*Silybum marianum*) is a natural polyphenol with high antioxidant and anti-cancer properties along with a few side effects (5- CELL JOURNAL(Yakhteh), Vol 16, No 3, Autumn 2014 300 12). Recent studies have shown the inhibitory effect of silybin in different cancers such as skin (13), colon (14), lung (15), prostate (16-17) and breast (18). Also, synergistic anti-cancer effects of silybin have been shown with other anti-cancer drugs such as doxorubicin (19), cisplatin and carboplatin (20) and mitoxantrone(21) in prostate cancers, and doxorubicin in MBA-MD-468 and MCF-7 breast cancer cell lines (22). The pharmacological activities assigned to silybin show that this phytochemical blocks VEGF, EGFR, COX-2 and TNF. Considering that a tumor cell uses multiple pathways to survive, drugs that intervene in a single pathway (e.g. Avastin) are unlikely to succeed. The advantage of plant-derived products, as described here, is that they intervene in multiple pathways. This characteristic supports the idea that they may have better anti-cancer potential. (23). However, the underlying mechanism of the inhibitory action of silybin in breast cancer has not yet been completely elucidated (5).

Also, recent *in vivo* studies on liver disease show that phosphatidylcholine bound to silybin is much more effective than silybin alone due to its bioavailability being 7 to 10 times more than silybin (24) Considering that bioavailability is influenced by a multitude of factors and has different levels including absorption, distribution (by the circulating blood), metabolism (by the liver), entry of the drug into specific body tissues, excretion and bioactivity, which in turn are governed by a large number of parameters (25, 26). However, in this *in vitro* study, certainly the bioavailability is only cell membrane absorption.

The absorption and therapeutic property of silybin is limited due to its poor water solubility (27) based on two factors. First, it is a multiplering molecule and too large to be absorbed by simple diffusion. Second, because it has poor miscibility with oils and other lipids of the membrane. Therefore the structure of silybin is limited in its ability to pass across the lipid-rich outer membranes of the enterocytes (intestinal absorptive cells) of the small intestine (28). Moreover, studies have shown that one of the multiple effects of silybin is the induction of growth inhibition and cell viability reduction in cancer cells (e.g. SHP-77 and A-549 lung carcinoma cell lines) (23). Hence, within this broader area, one specific research interest of ours was to evaluate cell viability reduction of T47D cancer cells by MTT, and to determin IC_50_ (half maximal inhibitory concentration) in order to estimate the comparative bioavailability of silybin with silybin-phosphatidylcholine.

In this study, we compared silybin with silybin- phosphatidylcholine in terms of cell membrane bioavailability, cytotoxicity and ESR expression (all by no serum starvation) in T47D human breast cancer cell line.

## Materials and Methods

### Tumor cell line and reagents

T47D is an ER^+^ human breast ductal carcinoma cell line. According to studies hitherto, it is not clear that T47D is a highly (29) or weakly (30) invasive (31- 33) or non-invasive (34, 35) cell line. A T47D cell line was purchased from the National Cell Bank, Pasteur Institute of Iran. The cell lines were cultured in RPMI1640 medium (Invitrogen) with 10% fetal bovine serum (FBS), 1% penicillin/streptomycin (all from PAA), 2 g/l sodium bicarbonate and 2.5 g/l HEPES (Sigma-Aldich, Missouri, USA). T47D cells were grown under standard culture conditions (37℃, 95% humidified air, and 5% CO_2_). For cell harvesting, 0.25% solution of trypsin (Sigma-Aldich, Missouri, USA) in PBS was used.

### Chemical treatments and MTT assay

For the MTT assay, the cells were first seeded in three 96-well microplates. In each well containing 100 µl complete medium, 7x10^3^ cells were seeded. The next day, the cells were treated with different doses of silybin (50, 75, 100, 150, 200, 250, 300, and 350 µM) or silybinphosphatidylcholine (50, 75, 100, and 150 µM) for 24, 48, and 72 hours. Our primary MTT tests showed that the cytotoxicity effects of silybinphosphatidylcholine are two or three times more than silybin, thus, some doses of silybin-phosphatidylcholine (i.e. 200, 250, 300, 350 µM) were not used. All doses were renewed every 24 hours. From the silybin (Sigma) stock solution, 100 mM was dissolved in dimethyl sulfoxide (DMSO). From the silybin-phoshphatidylcholine (Enzymatic Therapy, USA) stock solution, 10 mM was dissolved in DMSO: methanol at a ratio of 3:1. In all tests, the final concentration of DMSO did not exceed 0.1% (v/v).

After the 24, 48, and 72 hours treatments, the cells were incubated with 0.5 mg/ml microculture tetrazolium (Sigma) for about 3 hours. The optical density (OD) of formazan dye dissolved in DMSO was measured with an ELISA microplate reader (Gen5, Power Wave XS2, BioTek, USA) at 570 nm.

The percentage of cell viability at different doses was calculated by the following equation:

$$\mathrm{Cell viability percentage}=\frac{\mathrm{OD treated Well}}{\mathrm{OD control Well}}\times 100$$

### IC_50_ determination

The half maximal inhibitory concentration (IC_50_) of silybin and silybin-phosphatidylcholine was determined by using the Pharmacologic Calculation System statistical package (Pharm PCS) (Springer Verlag, USA) after 24, 48, and 72 hours in the T47D cell line.

After the MTT assay, and the determination of IC_50_, some doses were selected (75 μM and 150 μM for silybin, 25 μM and 50 μM for silybinphosphatidylcholine) for *ESR1* and *ESR2* gene expression analysis after 24, 48, and 72 hours. Each experiment had three individual samples (Error bars: ± SD).

### RNA extraction and cDNA synthesis

Cells were seeded in three 6-well microplates. 2×10^5^ cells were seeded in wells each containing 200 ml complete medium. After 24 hours, the cells were treated with 75 and 150 μM silybin and 25 and 50 μM silybin-phosphatidylcholine doses for 24, 48, and 72 hours.

Total RNA was isolated from the treated cells using the RNX Plus™ kit (CinnaGen, Tehran, Iran) according to manufacturer’s instruction.

For cDNA synthesis, 1000 ng of extracted RNA was reverse transcribed into cDNA according to the manufacturer’s protocol, using EDTA (CinnaGen), dNTP (CinnaGen), and random hexamer primer (Fermentas, Pittsburgh PA, USA), Reverse Transcriptase 10000 u (Fermentas), RiboLock RNase Inhibitor 2500 u (Fermentas), DEPC Water (CinnaGen).

### Analysis of gene expression by real-time PCR

For *ESR1*, *ESR2* and *GAPDH* (as a control), the following primer sets were purchased from Qiagen: *ESR1* (QT00044492), *ESR2* (QT00060641), and *GAPDH* (QT01192646).

For each reaction, 1 μl cDNA was added to a 9 μl reaction mixture containing 1 μl of related primers and 5 μl SYBR Green I Master Mix (QuantiFast SYBR Green PCR, Q204054), and run on a Real Time Thermo cycler (RotorGene 6000, Corbett Life Science, USA). The real-time PCR program was as follows: initial denaturation 95˚C for 5 minutes, denaturation 95˚C for 15 seconds, annealing temperature optimized from 60 to 61˚Cfor 25 seconds, extension 72˚C for 25 seconds, 35 cycles. The specificity of the PCR product was assessed by verifying a single peak in melting curve analysis.

All measurements were taken twice in duplicate and the average was used for further analysis. *GAPDH*, a housekeeping gene, was used as a control; the fold change of each target gene relative to *GAPDH* was calculated based on relative quantitation using the ΔΔCT method, calculated by the 2 ^–ΔΔCT^ relative expression formula.

### Statistical analysis

**Fig 1:**
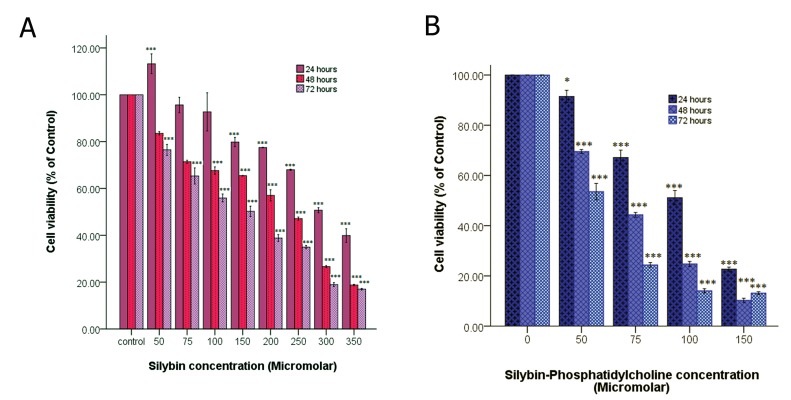
The effect of silybin (A) and silybin-phosphatidylcholine (B) on cell viability of the T47D breast cancer cell line. Data is presented as percentage of viability in three independent experiments. *; p< 0.05, **; p<0.01 and ***; p< 0.001.

**Fig 2:**
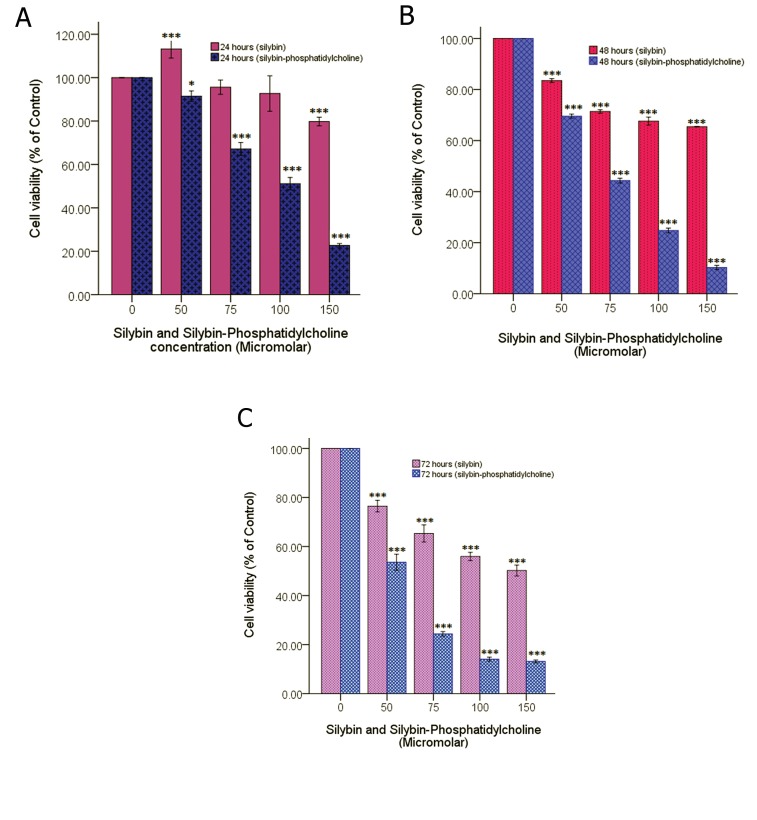
Comparison of different doses of silybin, and silybin-phosphatidylcholine after 24 (A), 48 (B), and 72 hours (C) of treatment. Data is presented as percentage of viability in three independent experiments. *; p< 0.05, **; p<0.01 and ***; p< 0.001.

Data were analyzed using SPSS 18 software. One-way ANOVA and Dunnett’s two-tailed post hoc t test were employed to evaluate the statistical significance of differences between the control and all treatments. The data had normal distribution. The P values that were considered significant are displayed as *; p<0.05, **; p<0.01, ***; p<0.001 in figures 1, 2, and 4. Cell viability graphs were depicted by SPSS 18 (clustered bar, summaries for group of case). The IC_50_s were estimated using the Pharmacologic Calculation System statistical package (Pharm PCS, Springer Verlag, USA).

**Fig 3:**
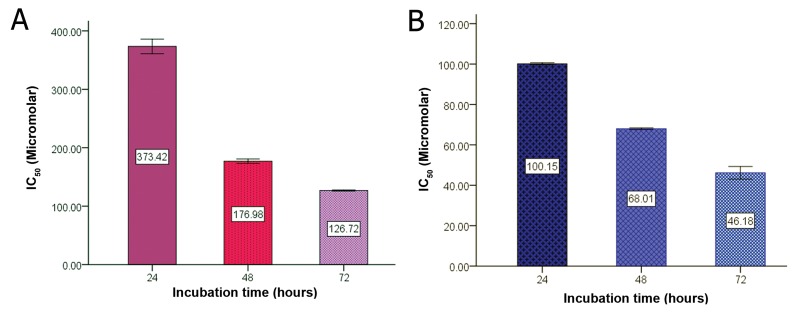
Determination of IC_50_ of silybin (A), and silybin-phosphatidylcholine (B) during 24, 48, and 72 hours of incubation.

**Fig 4:**
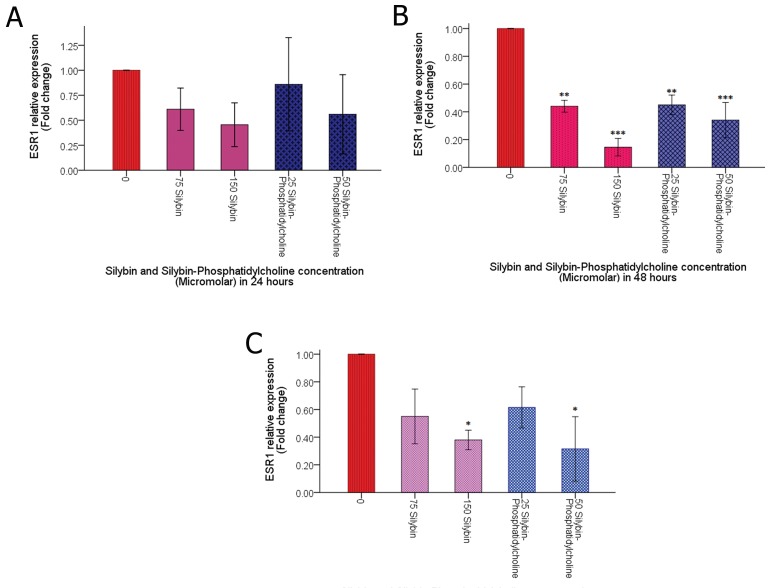
Effect of Silybin and Silybin-phosphatidylcholine on *ESR1* expression after 24 (A), 48 (B), and 72 hours (C) of treatment in the T47D breast cancer cell line. The reverse-transcribed RNA and amplified cDNA was normalized for *GAPDH* expression. Relative expression graphs were depicted by SPSS 18 (simple bar, summaries for group of case). Data were analyzed by the ΔΔCT relative expression method, and presented as two independent experiments. Each experiment had two individual samples (Error bars: ± SD). *; p< 0.05, **; p<0.01 and ***; p< 0.001.

## Results

### Proliferation and inhibitory effects of Silybin and Silybin-phosphatidylcholine on the T47D cell line

Briefly, 7×10^3^ cells were seeded in 96 well plates for 24 hours and treated with different doses in a complete medium (no serum starvation). The cytotoxicity effects of silybin and silybin-phosphatidylcholine were evaluated by MTT assay in the T47D cell line in eight doses (50, 75, 100, 150, 200, 250, 300, 350 μM), and four doses (50, 75, 100, 150 μM) respectively for 24, 48 and 72 hours (Fig 1). Cell growth inhibition was observed after 24, 48, and 72 hours of treatment. Silybin and silybin-phosphatidylcholine treatments resulted in a dose and time-dependent decrease in cell viability. However, increasing cell proliferation was observed in 50 μM silybin (low doses) in the first 24 hours.

The comparison of four doses of silybin and silybin-phosphatidylcholine (50, 75, 100, 150 μ higher M) after 24 hours of treatment shows that each silybin-phosphatidylcholine dose had a much higher inhibitory effect on cell growth than the same silybin dose (Fig 2A). As indicated, all doses except silybin 50 μM reduced cell proliferation, and all doses except 75 μM and 100 μM silybin were considered statistically significant (p<0.05 or p<0.001).

All doses after 48 and 72 hours of treatment decreased cell viability and were statistically significant (p<0.001). Figure 2B (after 48 hours of treatment) and figure 2C (after 72 hours of treatment) show that, each silybin-phosphatidylcholine dose had a much higher inhibitory effect on cell growth than the same silybin dose, and this difference was more significant in the 72 hours treatment than that of 48 hours.

Figure 3 shows the IC_50_s of silybin and silybin- phosphatidylcholine after 24, 48 and 72 hours of treatment. Data from three independent experiments are presented. The IC_50_ comparison of silybin and silybin-phosphatidylcholine indicated that the bioavailability of silybinphosphatidylcholine is 2.5-3 times more than silybin.

### Down regulation of *ESR1* gene expression after 24, 48, and 72 hours of treatment with Silybin and Silybin-phosphatidylcholine in the T47D cell line

According to the MTT assay results, silybinphosphatidylcholine is more effective than silybin on *ESR1* down-regulation. Thus, to compare the effect of these two compounds on *ESR1* and *ESR2* gene expression, the same doses were not used. Considering that silybin-phosphatidylcholine bioavailability in T47D cell line is 2.5-3 times greater than that of silybin, silybin doses were selected three times more than the silybinphosphatidylcholine doses. On the other hand, the aim of this step of the study was to analyze *ESR1* and *ESR2* gene expression by real-time RT-PCR (no cell mortality). Hence, all selected doses were less than the IC_50_s. Therefore, silybin at concentrations of 75 and 150 μM corresponded to 25 and 50 μM of silybin-phosphatidylcholine respectively.

Figure 4A shows that all silybin and silybinphosphatidylcholine doses down-regulate *ESR1* but not significant after 24 hours.

As shown in figure 4B, the level of *ESR1* down regulation of 25 μM silybin-phosphatidylcholine after 48 hours is nearly the same of its corresponding dose of silybin (75 μM). After 48 hours, 150 μM silybin seems more effective than its corresponding dose (50 μM silybinphosphatidylcholine) (p< 0.01, p<0.001).

Figure 4C indicates that after 72 hours of treatment, only the high doses of silybin (150 μM) and silybin-phosphatidylcholine (50 μM) showed significant *ESR1*down-regulation. Overall, the most down-regulation was observed using 50 μM of silybin-phosphatidylcholine. The T47D cell line showed negligible *ESR2* expression.

## Discussion

Considering the aim of the study, to obtain reliable results, the MTT assays and cell treatments were not done by serum starvation, and the medium were exchanged every 24 hours. The comparison of silybin and silybin-phosphatidylcholine by MTT assay (by no serum starvation) indicates that all silybin-phosphatidylcholine doses had a much larger inhibitory effect on cell growth (2.5-3 times more) than the same silybin doses in the T47D cell line. This difference became more significant as the duration of treatment increased. The results reported in this study show significant dose- and time-dependent cell growth inhibitory effects of silybin and silybin-phosphatidylcholine in T47D cells after 48 and 72 hours of treatment at all doses. Also, in our latest studies on silybin cytotoxicity on MDA-MB-231 breast (36), and PC-3 prostate cancer (37) cell lines, all silybin doses had growth inhibitory effects after 24, 48 and 72 hours of treatment.

According to the 24 hours MTT assay results in T47D cells, silybin and silybin-phosphatidylcholine cytotoxicity were effective at most (but not all) doses. Since an increase in cell proliferation was observed at 50 μM of silybin (low dose) in the first 24 hours, choosing very low doses of this compound (depending on the type of the cell line) can cause opposite results (cell growth is stimulated at low concentration) and, thus, may result in misleading conclusions.

Moreover, many researchers use serum starvation because it commonly leads to cell cycle arrest in the G0/G1 phase, and also has been used to arrest the G1 phase in cancer cells (38).

In our latest research, the comparison of silybin IC_50_s, using complete medium and serum starvation procedures in MDA-MB-453 or BT474 cell line, indicates that IC_50_ reported doses by serum starvation method are less than the complete medium method (data not shown). Therefore, for studies that are not focused on cell cycle arrest, to obtain reliable results, serum starvation method should not be used for cell treatments.

Breast cancer is a major public health problem worldwide and about 70% of primary breast tumors in women are ER-positive (ERα) (39). Phytochemicals such as flavonoids have good potential as anti-cancer agents because of their anti-proliferative activity against human tumor cell lines, safety and ability to target multiple cell-signaling pathways (40-41). Silybin is a flavonoid antioxidant that has been used as both an antihepatotoxic and an anti-carcinogenic agent (42). More importantly, it has been reported that silybin has no significant effect on the growth of normal human prostate epithelial cells (43). Siliphos was shown to be well tolerated in acute and long-term toxicity tests in rodents and primates up to oral doses of 2000 mg/ kg (as silybin). The excellent tolerability of this complex was confirmed in volunteers at doses up to 360 mg p.o. (as silybin) for three weeks (28). Phytosomes such as silybin-phosphatidylcholine are advanced forms of herbal formulations that are better absorbed, and as a result produce better bioavailability and therapeutic action than the conventional herbal extracts such as silybin (44).

We examined the effect of silybin and silybin- phosphatidylcholine on *ESR1* expression in T47D breast cancer cells by RT-PCR. In the first 24 hours, all doses showed no significant down- regulation in *ESR1* expression, perhaps demonstrating that for optimum effects of silybin and silybin-phosphatidylcholine on *ESR1* regulation, more than 24 hours of treatment is required. The results for 48 hours indicated all doses significantly down-regulated *ESR1* (p<0.01 or p<0.001). The results also showed that 75 μM silybin and 25μM silybin-phosphatidylcholine almost down-regulated *ESR1* as the same level, indicating that, the 25 μM silybin-phosphatidylcholine is as effective as 75 μM silybin which it is 3 times greater (three fold). The 72 hours treatment demonstrates 50 μM silybin-phosphatidylcholine down-regulates *ESR1* more than 75 and 150 μM silybin which are higher concentrations than the silybin- phosphatidylcholine dose. On the contrary, the T47D cell line showed negligible *ESR2* expression by real time RT-PCR, suggesting that this cell line is *ESR2* negative.

Some evidence has shown that anti-cancer drugs are not effective enough to treat all cases of cancers and may also show resistance (45, 46). For many years, tamoxifen was the mainstay of endocrine treatment for ER^+^ breast cancer (47), but recently the third-generation aromatase inhibitors (AIs) called estrogen receptor down- regulators (ERDs) such as fulvestrant (Faslodex) have started to be used ahead of tamoxifen in the first-line advanced (48) and adjuvant (49) settings because of their superior efficacy and tolerability profiles. Fulvestrant is an ER antagonist with no estrogen agonist effects and a novel mode of action; it binds, blocks, and increases degradation of ER (50).

Since cancer has different causes, and more than one mutation, sometimes blocking a receptor is not sufficient to silence the related cell signals (51, 52). Thus, flavonoids, such as silybin in contrast to some anti-cancer drugs (e.g. tamoxifen, fulvestrant) have more extensive effects on multiple cell signals, and may be used for different types of breast or other cancers. It can be used for a set period either singly or in combination with anti- cancer drugs to downregulate *ESR1*.

## Conclusion

This study suggests that silybin and silybinphosphatidylcholine down regulate *ESR1* in ER^+^ breast cancers. Results show that in T47D cells, silybin-phosphatidylcholine has a much higher inhibitory effect and down-regulated *ESR1* more significantly than silybin. However, systematic clinical trials are required to test silybin- phosphatidylcholine in order to fully understand its potential.
